# Image dataset for classification of diseases in guava fruits and leaves

**DOI:** 10.1016/j.dib.2025.111378

**Published:** 2025-02-07

**Authors:** Montasir Rahman Shihab, Nafiu Islam Saim, Mayen Uddin Mojumdar, Dewan Mamun Raza, Shah Md Tanvir Siddiquee, Sheak Rashed Haider Noori, Narayan Ranjan Chakraborty

**Affiliations:** Multidisciplinary Action Research Laboratory, Department of Computer Science and 8 Engineering, Daffodil International University, Birulia, Dhaka 1216, Bangladesh

**Keywords:** Guava, Disease, Agriculture, Image processing, Classification, Fruit

## Abstract

Guava (Psidium guajava) this is a tropical fruit and one of the common tropical fruits in Bangladesh. The economic and health value of this important crop is unmeasurable, but it quickly becomes infected with many diseases that can greatly reduce its yield and quality. Thus, the use of technology for automatic fruit and leaf disease detection is necessary in agriculture. The dataset provides us overall guava fruits & leaves image samples for detection of diseases in guava fruits and leaves. This dataset consists of images of healthy and diseased samples infected by fruit disease such as anthracnose, scab, styler end root and leaf disease such as canker, rust, anthracnose and dot. It consists of 3,432 real images obtained from different places in Bangladesh. It is also extended 20,344 augmented images ready to be used for machine learning purposes. This dataset serves as a fundamental building block for utilizing machine learning and computer vision techniques to develop automated detection systems of various diseases. It assists in the early detection of diseases affecting guava, and provides them with solutions to intervene there itself saving agricultural yield and nutritional losses while also promoting sustainable farming practices. This dataset will assist researchers for progressing guava detecting disease through the execution of computational models and application of better machine learning techniques.

Specifications Table

**Table:** 

Subject	Computer science, Agricultural
Specific subject area	Image classification, machine learning, computer vision, Image processing
Type of data	.JPG Image
Data collection	The dataset image of guava fruits and leaves were captured with Iphone 15 , Realme gt master and redmi note 10 camera .the images were captured with proper distance .the guava fruits and leaves were first collected from guava garden in Cumilla and Fruit market in Dhaka .the samples are placed on a white paper and took different photos from different angle.
Data source location	1. Rampur,Burichung,Cumilla,Bangladesh (Latitude: 23°31′50.2″N , Longitude: 91°05′09.1″E) 2. Asulia Bazar, Dhaka, Bangladesh (Latitude: 23.8971° N , Longitude: 90.3309° E) 3. Baipayl, Dhaka, Bangladesh (Latitude: 23°56′44.0″N Longitude: 90°16′27.3″E)
Data accessibility	Repository name: Mendeley Data Data identification number: 10.17632/fspx44mwfp.1 Direct URL to data: https://data.mendeley.com/datasets/fspx44mwfp/1

## 1 Value of the Data

• The Guava Leaf and Fruit Disease Dataset contains images of healthy guava leaves and fruits and also affected with various diseases such as fruit anthracnose, scab, styler root end, and leaf canker, dot, rust, and anthracnose. This comprehensive dataset allows to develop and train models to detect and classify different disease.
• The dataset plays a crucial role in automating the process of early guava disease identification. By enabling early detection through machine learning models, it helps farmers address issues before they escalate, leading to improved crop management, reduced losses, and more sustainable farming practices.
• The dataset is a valuable resource for advancing research in machine learning, computer vision, and plant pathology. It facilitates the development of algorithms and models to accurately identify and classify guava diseases.
• The dataset provides valuable insights into various guava fruit and leaf diseases, serving as a foundation for further research and advancements. It enables the exploration of improved disease management and detection techniques.
• This dataset is useful for researchers and students to gain practical experience with real-world problems in plants fruits and leaves diseases and utilize better management techniques for that.

## 2 Background

Guava (*Psidium guajava*), a tropical fruit rich in nutrients and antioxidants, is cultivated worldwide for its economic and health benefits [1] Guava has its nutritional benefits and wide medicinal uses, including anti-diarrheal, antihypertensive, and antimicrobial properties. Its fruit and leaves contain beneficial compounds like quercetin and flavonoids, which have shown promising effects in human trials. The plant is considered safe and has significant therapeutic potential for various ailments [2,3]. This fruit is adaptable to poor soils but highly susceptible to diseases caused by various pathogens, including fungi, bacteria, algae, nematodes, and physiological disorders. These diseases affect multiple parts of the plant, such as fruits, leaves, twigs, and roots, leading to significant pre- and post-harvest losses. Key diseases include wilt, anthracnose, canker, scab, various fruit rots, leaf blight, and rust. Additionally, deficiencies in nutrients like zinc and magnesium, along with physiological issues such as internal breakdown, contribute to reduced guava productivity [4]. By detecting these common diseases , we can reduce agricultural and nutritional losses in guava and improve its productivity .The dataset by A. Rajbongshi (2022) [5] titled “A comprehensive guava leaves and fruits dataset for guava disease recognition” consists of 681 samples of disease affected, and disease-free fruit and leaf can be used for disease detection but limitation in some disease types .The dataset by A. S. M. Farhan Al Haque (2019) [6], titled, “A Computer Vision System for Guava Disease Detection and Recommend Curative Solution Using Deep Learning Approach’’ contains 4 classes 3 classes of diseases like anthracnose, canker, fruit rot and 1 healthy fruit samples each contain 2500 samples used for disease detection limitation in fruit disease and leaf samples. The dataset by PATHMANABAN P (2023) [7], contains 3 classes healthy samples (600 images), damaged samples (1100 images),and diseased samples (395 images), which are further categorized into specific disease types such as wilt, anthracnose, canker, and rot but there's is no leaf samples. Our dataset offers an extensive collection of guava fruit and leaf disease samples, encompassing a wide range of disease classes. It comprises 3432 original images and an additional 20,344 augmented images, providing a robust resource for effective disease detection and classification.

In Table 1, a comprehensive understanding of the current work dataset, among other dataset. There is a variety healthy and disease of fruits and leaves classes by which you can readily comprehend the significance of this dataset.

**Table 1:** Comparison with existing datasets.

SL	Classes	Our Dataset	A. Rajbongshi [5]	PATHMANABAN P [7]	A. S. M. Farhan Al Haque [6]
1	Healthy (Fruit)	✓ (470)	✓	✓	✓
2	Anthracnose (Fruit)	✓ (263)	X	✓	✓
3	Scab (Fruit)	✓ (119)	✓	✓	X
4	Styler end rot (Fruit)	✓ (262)	✓	✓	✓
5	Healthy (Leaf)	✓ (1498)	✓	X	X
6	Anthracnose (Leaf)	✓ (237)	X	X	X
7	Dot (Leaf)	✓ (219)	X	X	X
8	Canker (Leaf)	✓ (192)	X	X	X
9	Rust (Leaf)	✓ (167)	✓	X	X

A comparison between sample images from our Guava Leaf and Fruit Disease Dataset with other existing available datasets is shown in Table 2. As you can see from the table that our data is preprocessed and removed unnecessary background objects. This makes data easier to focus on the features and gain insights from the images. The compared image datasets offer limited classes like image dataset by A. K. Maitlo, PATHMANABAN P have only fruit samples and A. Rajbongshi have fruits and two leaf class but our dataset provided more variety of fruit and leaf classes. The detailed quality of the dataset makes it well prepared for furthermore study and analysis.

**Table 2:** Comparison with existing image dataset.

## 3 Data Description

The Guava Leaf and Fruit Disease Dataset contains a total of 3432 guava fruit and guava leaf images, collected from various sources to ensure diversity and representativeness in the dataset. Devices like Realme GT Master, Redmi Note 10, and iPhone 15 were used to take the pictures. The photos are in are in JPG format and the resolution of the images 3024×4032 pixels at dpi 96. The fruit images are divided into two categories: healthy and diseased, with the diseased category further subdivided into three types of diseases: anthracnose, Scab, and Styler Root end with 1119 guava fruits collected from guava garden and fruit market and , while the remaining 2313 guava leaf images consisting healthy and disease like anthracnose, canker, rust, dot were collected from garden providing a natural representation of the leaves in various conditions. The statistics of the dataset is given in Table 3.

**Table 3:** Statistics of the Guava fruits and leaves Dataset.

Classes Name	Original images	Augmented images
Healthy (Fruit)	470	4230
Anthracnose (Fruit)	263	2367
Scab (Fruit)	119	1190
Styler end rot (Fruit)	262	2358
Healthy (Leaf)	1498	4494
Anthracnose (Leaf)	237	1659
Dot (Leaf)	219	1533
Canker (Leaf)	192	1344
Rust (Leaf)	167	1169

In Table 4 presents a comparison between healthy and diseased guava fruits, detailing the distinguishing features and characteristics observed in each category to classification. It highlights visual differences for effective identification.

**Table 4:** Guava fruits description.

Name	Description	Image
Healthy	Fresh guavas have an oval shape and smell like lemon peel. Depending on species, their skin can either be thick and bitter or soft and sweet when they ripen, from which the color changes from green to maroon yellow or stays green [8].	
Anthracnose	This fruit of guava, common fungal disease caused by *Colletotrichum*, with inky dark lesions and its spore mass. The lesions expand and penetrate the pulp of the fruit, leading to substantial postharvest losses [9].	
Scab	Scab is a severe fungal disease that causes corky, round lesions on guava fruits, reducing quality and market value. It primarily affects green fruits, producing brown scabby patches, especially under high humidity. The disease softens peels, leading to decay, particularly in fruits on the ground [5].	
Styler end root	This disease, caused by the ascomycete fungus *Phomopsis*, primarily presents as discoloration beneath the persistent calyx of the fruit. Over time, this area gradually expands, becoming dark brown and soft [10].	

In Table 5 presents a comparison between healthy and diseased guava leafs, detailing the distinguishing features and characteristics observed in each category to classification. It highlights visual differences for effective identification.

**Table 5:** Guava Leaf description.

Name	Description	Image
Healthy	Those leaves are evenly green, glossy and even-structured with no spots, lesions or discolorations. The margins are whole, and the faces of leaves show no blemishes having typical features of healthy guava leaf.	
Anthracnose	Guava leaf anthracnose, caused by *Colletotrichum* species, manifests as dark, sunken lesions on leaves and occasionally stems. It can cause defoliation, lower photosynthesis efficiency, and weaken the plant's overall health [11].	
Dot	Dot disease appears as small, circular spots or lesions on the leaves, often caused by fungal infections. The spots may be brown, yellow, or black, and over time, they can cause the leaf tissue to die.	
Rust	Rust is a fungal disease characterized by reddish or orange spots on the lower sides of leaves, which develop into pustules. These pustules release spores which then infect other parts of the plant. Rust reduces its photosynthetic capacity and predisposes a plant to other diseases [12].	
Canker	Canker is a leaf fungus disease that will attack a guava leaves, which could kill these affected tissues. The lesions are most often depressed and red, brown or dark. The plant is compromised by stopping the photosynthetic channel that leads to it, and under extreme situations this may cause defoliation [4].	

Data collection was conducted during October and November 2024. Leaf images were captured on different days and at various times of the day across multiple locations. The details are provided in Table 6.

**Table 6:** Collection guava fruit and leaf samples.

Guava fruits and leaves	Weather	Date	Time	Device	Location
Healthy (Fruit)	Cloudy	5 November	Afternoon	iphone 15 and realme gt master	Baipayl,Dhaka (Latitude: 23°56′44.0″N Longitude: 90°16′27.3″E)
Anthracnose (Fruit)	Sunny	30 October	Noon	iphone 15 and realme gt master	Asulia Bazar,Dhaka (Latitude: 23.8971° N , Longitude: 90.3309° E)
Scab (Fruit)	Sunny	30 October	Noon	iphone 15 and realme gt master	Asulia Bazar,Dhaka (Latitude: 23.8971° N , Longitude: 90.3309° E)
Styler end rot (Fruit)	Sunny	30 October	Noon	iphone 15 and realme gt master	Asulia Bazar,Dhaka (Latitude: 23.8971° N , Longitude: 90.3309° E)
Healthy (Leaf)	Sunny	25 October	Morning	iphone 15 and redmi note 10	Ramupur,burichung, Cumilla (Latitude: 23°31′50.2″N , Longitude: 91°05′09.1″E)
Anthracnose (Leaf)	Sunny	26 October	Morning	iphone 15 and redmi note 10	Ramupur,burichung, Cumilla (Latitude: 23°31′50.2″N , Longitude: 91°05′09.1″E)
Canker (Leaf)	Sunny	26 October	Morning	iphone 15 and redmi note 10	Ramupur,burichung, Cumilla (Latitude: 23°31′50.2″N , Longitude: 91°05′09.1″E)
Dot (Leaf)	Sunny	26 October	Afternoon	iphone 15 and redmi note 10	Ramupur,burichung, Cumilla (Latitude: 23°31′50.2″N , Longitude: 91°05′09.1″E)
Rust (Leaf)	sunny	26 October	Noon	iphone 15 and redmi note 10	Ramupur,burichung, Cumilla (Latitude: 23°31′50.2″N, Longitude: 91°05′09.1″E)

## 4 Experimental Design, Materials and Methods

### 4.1 Preprocessing

**Fig. 1:**
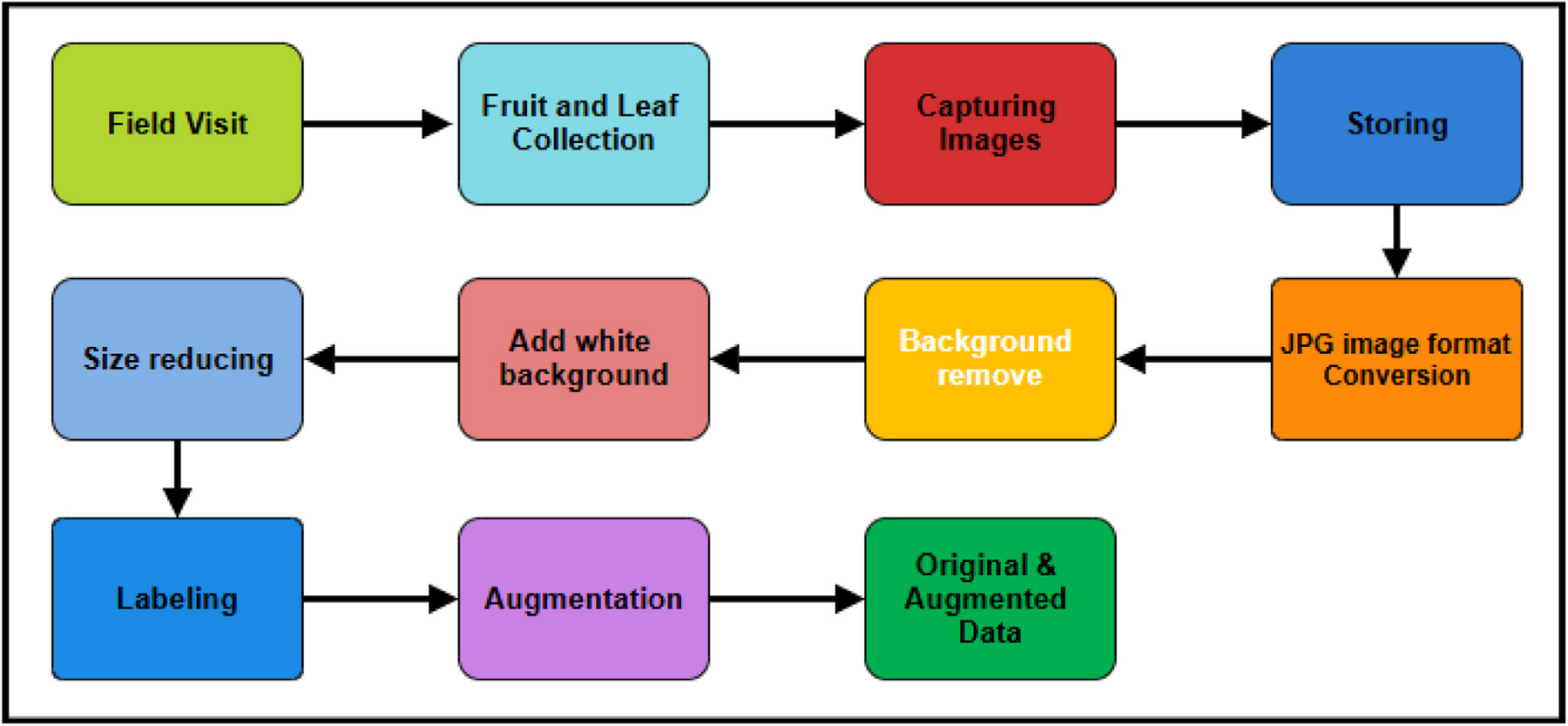
Basic steps of data workflow.

The processes we used to create a comprehensive and distinctive dataset are depicted in Fig. 1. We began by identifying significant illnesses and seeking advice from specialists. A comprehensive pre-processing workflow was executed to standardize the dataset and optimize it for machine learning tasks, including resizing, data augmentation.

The preprocessing for the dataset starts with field visits where we collect both healthy and diseased guava fruits and leaves. These images are captured under natural lighting to ensure clarity and minimize environmental interference like shadows or reflections. Once the images are gathered, they are stored for easy access and management. Next image format conversion was done for the iPhone 15 images, which were initially in HEIC format. This images were later converted to JPG using Pythons Pillow library and pillow-heif plugin, while the other images from the Redmi Note 10 and Realme GT Master were already in JPG format. The next step involves background removal using the rembg library, which isolates the fruit or leaf images by removing the background. The image is then transformed to an RGBA type where transparent parts are replaced by a single-color white background, so that all images have the same appearance. Then the image size is reduce the image dimension to how it can be working for teaching machine learning models [13].

The images are all labelled with respect to their condition such as Healthy and disease-affected. For labelling the images below steps were taken:

First Inspection: Every picture was carefully inspected to determine its quality and make sure it captured the features of the condition under study.

**Fig. 2:**
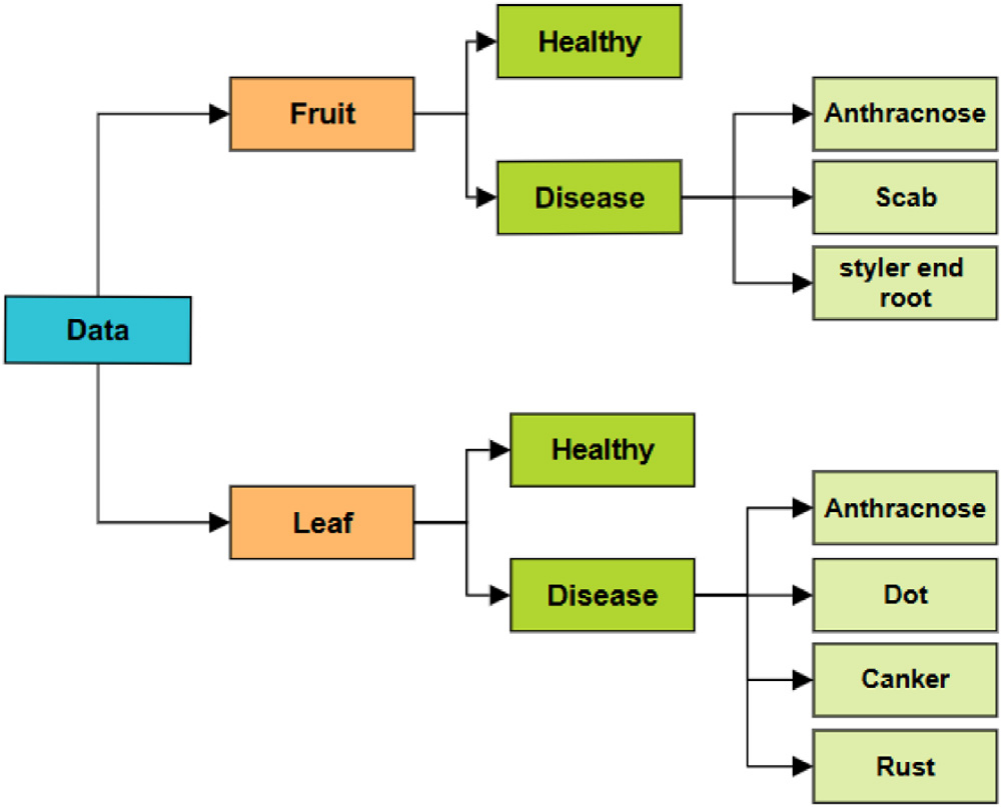
Image labeling structure.

Assigned to Class: The images were separated into classes of fruit and leaves, which were further separated into healthy and diseased categories. The disease classes were then categorized into the particular diseases shows in Fig. 2.

Verification: After the first labeling, the annotations were checked to make sure they were accurate and consistent throughout the dataset. Agronomic expert Abdul Mannan Mojumdar, who works as an Additional Director at Bangladesh's Department of Agricultural Extension, assisted with the annotation.

Lastly, augmentation methods are used to create additional variability in the dataset. In order to improve diversity by mirroring the samples, the augmentation method starts with a horizontal flip applied to the photos. In order to accommodate for fluctuations in lighting conditions, random brightness and contrast adjustments are then added. To simulate various orientations, the photos are then randomly rotated range of 40°. Then, to add smoothness and lower noise, Gaussian blur is used with a kernel size that varies between limit (3,7). After that, a random scaling operation is executed, changing the image sizes within a 20 % range. Lastly, there is a one of the following transformations will be chosen at random brightness and contrast adjustments include embossing to create a raised or sunken impression, sharpening to accentuate edge details. To create a varied and rich dataset and enhance the model's resilience and generalization skills [14,15], shown in Fig 2.

**Fig. 3:**
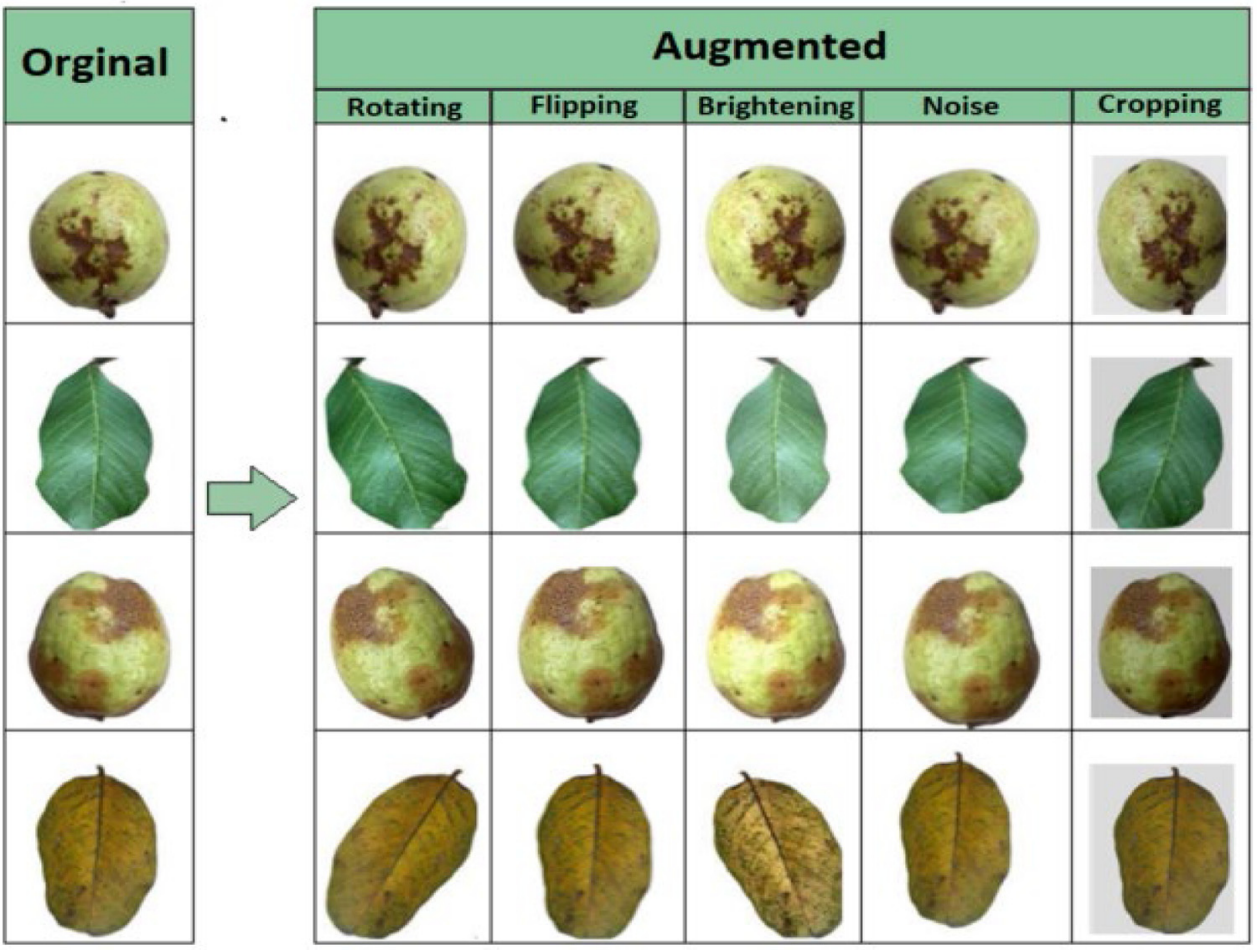
Sample augmented images that are produced from original images.

As a result, the original 3432 images were augmented, expanding the dataset to 20,433 images, which will provide a more comprehensive dataset for model training. Fig 3

## Limitations

In the dataset some categories have fewer samples due to limited availability. The image quality of some sample may be not good enough because of taking too much picture with in short period of time. The dataset is collected from specific place there is a geographic bias . Classification model can be apply with pre-trained models [16].

## Ethics Statement

The dataset utilized in this study was gathered in an ethical manner, guaranteeing that no plants, animals, or the environment were harmed in the process. The photos were appropriately procured, and when necessary, the appropriate permissions were acquired. The dataset is only meant for scholarly and research uses, with the goal of advancing the identification and control of agricultural diseases.

## CRediT Author Statement

**Montasir Rahman Shihab:** Conceptualization, Methodology. **Nafiu Islam Saim:** Data curation,Writing. **Mayen Uddin Mojumdar:** Supervision, Methodology. **Dewan Mamun Raza:** Data Curation. **Shah Md Tanvir Siddiquee:** Visualization. **Sheak Rashed Haider Noori:** Software. **Narayan Ranjan Chakraborty:** Writing – Review.

## Data Availability

Mendeley DataImage Dataset for Detection and classification of Diseases of Guava Fruits and Leaves (Original data).
